# Communication-Based Teaching on Childhood Obesity and the Planetary Health Diet in Medical Education: Proof-of-Concept Study Comparing 4 Information Sources

**DOI:** 10.2196/92644

**Published:** 2026-05-08

**Authors:** Teresa Festl-Wietek, Carla Schröpel, Friederike Holderried, Bernd Herrmann, Jan Griewatz, Stefan Ehehalt, Florian Junne, Stephan Zipfel, Anne Herrmann-Werner, Rebecca Erschens

**Affiliations:** 1 TIME - Tübingen Institute for Medical Education, University of Tübingen, Elfriede-Aulhorn-Straße 10, Tübingen, 72076, Germany, 49 70712973715; 2 Department of Psychosomatic Medicine and Psychotherapy, University Hospital Tübingen, University of Tübingen, Osianderstraße 5, 72076 Tübingen, Germany, Tübingen, Germany; 3 German Center for Mental Health (DZPG), partner site Tübingen, Tübingen, Germany; 4 Public Health Department of Stuttgart, Stuttgart, Germany; 5 Department of Psychosomatic Medicine and Psychotherapy, Otto von Guericke University Magdeburg, University Hospital Magdeburg, Magdeburg, Germany

**Keywords:** planetary health, medical education, obesity, patient-physician communication, artificial intelligence, AI

## Abstract

**Background:**

Childhood obesity constitutes a complex medical and psychosocial challenge that requires both nutritional knowledge and sensitive, relationship-oriented doctor-patient communication. The Planetary Health Diet links individual health promotion with environmental sustainability and represents a relevant framework for contemporary medical education.

**Objective:**

This proof-of-concept study investigated how different information sources influence medical students’ acquisition, structuring, and application of knowledge on childhood obesity and the Planetary Health Diet within a communication-based teaching setting, including the exploratory use of artificial intelligence–based tools.

**Methods:**

A total of 359 second-year medical students participated in a mandatory communication seminar during the 2023‐2024 academic year. Following a precourse knowledge assessment and a brief theoretical introduction, students worked on a standardized counseling scenario addressing childhood obesity. In small groups, students used only 1 assigned information source (ChatGPT, Google Search, scientific papers, or instructional videos) to prepare a counseling approach. Group outcomes were assessed using a predefined scoring system based on a sample solution, complemented by thematic content analysis.

**Results:**

All information sources enabled students to acquire relevant knowledge on childhood obesity and the Planetary Health Diet. However, groups differed with regard to the depth, differentiation, and structuring of their responses. The ChatGPT group achieved the highest conformity scores with the sample solution and provided the most additional information, followed by the Google and video groups, while the paper group achieved the lowest scores. Prior to the course, students reported limited knowledge of the Planetary Health Diet and little practical experience in counseling children with obesity and their families.

**Conclusions:**

Communication-based teaching formats provide an effective framework for introducing medical students to complex topics such as childhood obesity and sustainability-related nutrition early in their training. Easily accessible digital tools, including artificial intelligence–based systems, may facilitate knowledge acquisition and elaboration; however, their use requires explicit didactic framing, critical source evaluation, and reflection on the complexity of chronic conditions to support responsible and realistic learning outcomes in future physicians.

## Introduction

### Background

Physicians increasingly confront nutrition-related issues in clinical practice, particularly given the rising prevalence of childhood overweight and obesity [1]. Childhood overweight and obesity represent a major challenge for health care systems worldwide [2,3]. In Germany, approximately 15% of children and adolescents aged 3 to 17 years are affected by overweight, including obesity [4]. Evidence suggests that overweight and obesity often persist from childhood into adulthood, thereby increasing the risk of concomitant and secondary physical and mental health conditions [5-7]. At the same time, weight normalization can improve health outcomes [8,9]. Current recommendations emphasize that obesity treatment should be embedded in a multimodal approach combining physical activity, behavioral strategies, and dietary interventions [10].

One dietary framework that has gained increasing attention is the Planetary Health Diet [11]. The Planetary Health Diet refers to a predominantly plant-based reference diet for adults that aims to optimize human health and environmental sustainability within planetary boundaries [11]. Beyond individual health, it considers the ecological footprint of dietary behaviors and links health promotion with environmental sustainability [12]. In a similar direction, the EAT-Lancet Commission proposed a global strategy for agriculture and nutrition to protect both human health and planetary boundaries [11]. The strategy recommends increasing the intake of fruits and vegetables, roughly doubling the consumption of legumes and nuts, and reducing the consumption of meat and sugar, thereby outlining a dietary pattern that may support weight normalization while also addressing sustainability-related concerns. Based on this dietary pattern, the Planetary Health Diet may offer a useful framework for addressing childhood obesity because it promotes a predominantly plant-based diet, which is in accordance with the German nutrition guidelines for children [13].

Given their relevance for current and future health care, childhood obesity and the Planetary Health Diet should be addressed in medical education [14,15]. Nutrition and the reduction of overweight are becoming increasingly important in the management of a broad range of diseases, from metabolic disorders to mental health problems. Therefore, medical students need to acquire relevant knowledge during medical training and develop the skills required to translate this knowledge into effective patient counseling. Communication-based teaching, including clinical encounters and counseling about nutrition and weight-related concerns, requires not only biomedical knowledge but also sensitive, patient-centered communication, including awareness of psychosocial stress, stigma, and family dynamics [16]. Integrating the Planetary Health Diet into medical training may provide an opportunity to connect nutritional counseling with broader preventive perspectives and to foster self-management and lifestyle-related counseling skills within medical interviews [17,18]. Promoting healthy lifestyle behaviors and supporting patients’ self-management play a decisive role in such consultations [19].

Despite its clinical relevance, medical students have reported feeling insufficiently prepared to address childhood obesity and have expressed a need for additional training [15,20]. Prior work suggests that obesity and nutrition education should combine observation with supervised practice to build confidence and competence in counseling [20]. Communication-based teaching formats, such as counseling, may be particularly suitable for this purpose, as they allow students to work with realistic scenarios and practice how to translate knowledge into patient-centered recommendations. Furthermore, several skills, such as retention, empathic behavior, and knowledge transfer, can be trained in communication-based teaching formats, and differences in how these skills are acquired may affect students’ ability to apply their knowledge effectively and engage in patient-centered communication.

Medical students draw on various information sources when preparing for clinical tasks. Alongside traditional materials such as scientific papers and educational videos, internet-based searching has become commonplace [21]. More recently, artificial intelligence (AI)-based tools such as ChatGPT are increasingly being used as easily accessible sources of information [22]. While such tools may support rapid knowledge acquisition and elaboration, their use also raises didactic questions regarding information quality, source transparency, and students’ critical evaluation skills. To date, different information sources have not been systematically compared within a communication-based didactic context on childhood obesity and the Planetary Health Diet in medical education.

### Aim

This study aims to investigate medical students’ perspectives on childhood obesity and the Planetary Health Diet. Furthermore, this proof-of-concept study addresses the exploratory research question of whether and how four didactic scenarios within a communication seminar differ in (1) supporting medical students’ knowledge acquisition, (2) knowledge structuring, and (3) communication-related preparation for counseling interviews on childhood obesity and the Planetary Health Diet.

## Methods

### Study Design

This proof-of-concept study examined 4 didactic scenarios using different scientific and digital information sources within a communication-oriented medical education setting. The aim was to assess medical students’ perspectives on childhood obesity and the Planetary Health Diet and then compare their effectiveness in supporting knowledge acquisition and communication-related preparation on the topics of childhood obesity and the Planetary Health Diet in the context of counseling interviews. The comparison of these outcomes was intended to capture different but interrelated stages of learning that are relevant for counseling competence: acquiring knowledge, organizing it meaningfully, and translating it into communication-oriented preparation.

The study was conducted during the Winter Semester of the 2023‐2024 academic year (6 courses) and the Summer Semester of 2024 (7 courses) as part of a well-established communication course at the Medical Faculty of the University of Tübingen [23]. Approximately 30 second-year medical students were enrolled in each course. All second-year medical students attending the course were eligible to participate. Participation in the study was voluntary, while attendance at the teaching session itself was mandatory. The students were randomized into 4 different groups.

### Ethical Considerations

The study received ethical approval from the ethics committee of the Faculty of Medicine at the University of Tübingen (003/2024BO2) and was conducted in accordance with the Declaration of Helsinki in its current version (2024). Participation in the study was voluntary and had no impact on students’ academic standing, while attendance in the course itself was mandatory. No incentives or reimbursements were provided.

Data collection was conducted anonymously, and no personally identifiable information was recorded. In accordance with local ethics regulations, written informed consent was not required, as the study focused exclusively on the evaluation of didactic strategies and the generation of educational knowledge. Students were informed that materials produced during the course would be analyzed for quality assurance and educational research purposes and could be published in scientific contexts. Under German regulations, written consent is required only for epidemiological research involving personally identifiable data (Berufsordnung für Ärzte in Baden-Württemberg, § 15 Abs. 1) [24].

### Procedure and Course Content

The course content was developed by an interprofessional team of health care professionals with expertise in psychosomatic medicine, communication, and sustainability-related health education. At the beginning of the session, students’ pre-existing knowledge was assessed using a 10-minute questionnaire administered via Mentimeter [25], comprising 8 questions related to childhood obesity and the Planetary Health Diet.

Subsequently, students received a 10-minute theoretical introduction to counseling interviews delivered by experienced lecturers, followed by a 5-minute video illustrating a standardized consultation scenario. The video depicted a consultation following a school entrance examination at a district office, in which a physician informs a father that his son has childhood obesity. In response, the father expresses insecurity and psychosocial stress and asks for guidance and possible courses of action.

After viewing the video, students were divided into 4 groups. During a 5-minute instruction phase, they were tasked with preparing a counseling approach for the father that addressed the application of the Planetary Health Diet as part of obesity management, while using open, empathetic, and patient-centered communication. During a 30-minute group work phase, each group was required to gather and structure information using only one assigned information source: the ChatGPT group used GPT-3.5 [26], the Google group used Google Search, the paper group used preselected scientific papers [1,11,16,19], and the video group used provided scientific educational videos. Specifically, a video on planetary health and dietary transformation (health aspects of plant-based nutrition and required changes in food systems; relevant section: 00:33:48-00:50:30) [27] and an established educational video on childhood obesity (covering causes, consequences, and dietary treatment options) [28] were provided.

The group work was supervised by a lecturer to ensure adherence to the task and timeframe. The predefined video, provided paper, open internet search (Google), and AI-generated output (ChatGPT) were not intended to represent equivalent media formats; rather, they were compared as different but educationally relevant information resources that students could use to prepare for the same communication-based task.

**Figure 1.**
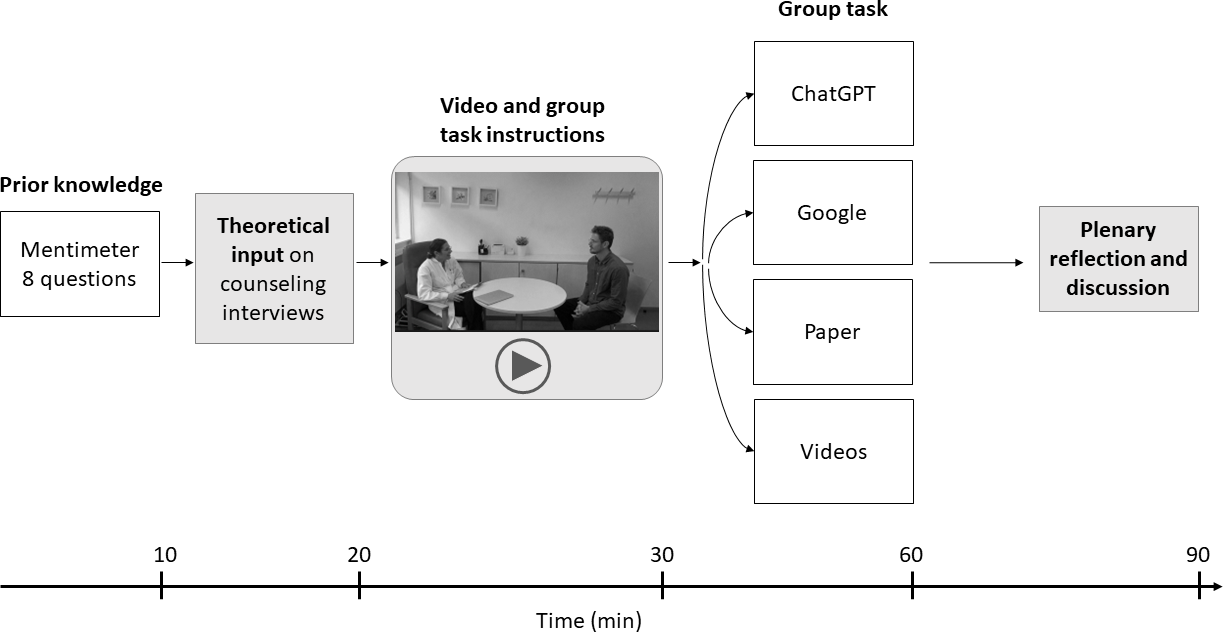
Overview of the course structure and sequence of teaching activities for second-year medical students participating in the counseling training on childhood obesity and the Planetary Health Diet. The diagram shows the sequence of the lesson components and their duration. The lesson lasted 90 minutes. The course was developed by an interprofessional team and included an initial Mentimeter questionnaire to assess students’ prior knowledge of childhood obesity and the Planetary Health Diet, followed by a theoretical introduction and a consultation video. Students then worked in 4 groups to prepare a counseling approach using 1 assigned information source (ChatGPT [GPT-3.5], Google Search, preselected scientific papers, or educational videos), followed by a moderated plenary discussion.

Following the group work, all students reconvened in a plenary session to present, discuss, and reflect on their findings and approaches during a 30-minute moderated discussion. An overview of the course structure and timing is provided in Figure 1.

### Mentimeter Questionnaire on Medical Students’ Perspectives

As part of the theoretical introduction to counseling interviews, students completed a brief questionnaire administered via Mentimeter [25] to assess pre-existing knowledge, attitudes, and experiences related to childhood obesity and the Planetary Health Diet. The questionnaire consisted of 8 items.

Questions 1‐7 assessed students’ perceptions, experiences, and attitudes and were rated on a 5-point Likert scale ranging from 1 (“not important at all/no experience at all/do not agree at all”) to 5 (“very important/a lot of experience/totally agree”). Question 8 was open-ended and aimed to capture students’ spontaneous associations with the Planetary Health Diet.

The questionnaire items were as follows:

- How important do you personally think it is to understand and treat childhood obesity?
- Have you had any practical experience with children with obesity and their parents during your medical training?
- Do you think that children with overweight and obesity are more stigmatized because of their weight?
- How important do you think it is for doctors and medical staff to have adequate knowledge of childhood obesity?
- A Planetary Health Diet can help children with obesity.
- “I try to eat a climate- and environmentally-friendly diet (eg, plant-based, regional, seasonal, organic).”
- It is important to inform patients about planetary health and Planetary Health Diets.
- What do you associate with the Planetary Health Diet?

### Group Task to Assess Knowledge Acquisition

Each of the 4 groups received an identical exercise sheet describing a standardized counseling scenario. Students were asked to imagine taking on the role of the physician from the video (Dr Johner) and conducting a short consultation with Mr Kleber regarding his son, Leon, who had been identified as having childhood obesity. Working in small groups, students were instructed to develop the content of the counseling conversation. They were provided with materials related to early childhood obesity and the Planetary Health Diet and were asked to gather and structure relevant information. In addition, students were explicitly instructed to consider interactional aspects and to apply medical dialogue techniques appropriate to the counseling context.

Crucially, each group was required to use only 1 assigned information source to complete the task: ChatGPT, scientific videos, preselected scientific papers, or Google Search. In addition to content-related aspects, students were asked to reflect on interactional considerations relevant to the counseling interview.

All groups were required to answer the same 5 content-related questions as follows:

- What is the definition of childhood obesity?
- What are the main causes of obesity in children?
- What are some long-term consequences of childhood obesity?
- What dietary guidelines should be followed for childhood obesity?
- What does the Planetary Health Diet look like, and what aspects does it take into account?

At the end of the group task, all groups submitted their written responses. In addition, students in the ChatGPT group submitted the saved chat history for further qualitative analysis.

### Data Analysis

Quantitative data analysis was conducted using Microsoft Excel. Descriptive statistics were calculated, including means, SDs, frequencies, and percentages.

To evaluate the content-related performance of the group task, a predefined sample solution for 5 questions was developed by a team of subject-matter experts (Table 1). These authors independently reviewed the questions and proposed relevant answer categories and scoring criteria, which were subsequently discussed and refined with the involvement of additional coauthors. For each question, up to 2 points were awarded when the group’s answer matched the sample solution (“conformity with sample solution” score), resulting in a total possible score ranging from 0 to 130 points across all courses. In addition, correct information that exceeded the sample solution was documented and quantified as “additional correct information,” with 0.5 points awarded per additional correct aspect. All group responses were evaluated independently by 2 raters (TFW and CS). In cases of discrepancies, a third rater was consulted (RE). The analysis was primarily descriptive. Groups were compared with respect to the (1) “conformity with sample solution” score and (2) the number of additional correct information provided. No inferential statistical tests were performed, as the aim of the analysis was to provide a descriptive comparison of group performance rather than to test statistical hypotheses. All groups submitted their written responses, and no missing data occurred.

**Table 1.** Sample solutions for 5 questions in the group task, as developed by an expert panel^a^.

Question	Sample solution^b^	Example of additional correct information^c^
(1)	BMI ≥95th or >97th percentile^d^ Age and gender taken into account	Skinfold measurement and waist circumference
(2)	Psychological factors (eg, boredom, stress, emotional eating, and family functioning) Unhealthy diet and lack of exercise	Genetic causes and medication
(3)	Physical consequences (at least 1 example; additional examples scored as additional information) Psychological consequences	Diabetes and orthopedic problems
(4)	Healthy eating such as vegetables (at least 1 example; additional examples scored as additional information) Eating behavior such as regular family meals (at least 1 example; additional examples scored as additional information)	No sugary drinks
(5)	Sustainability, environmental health promotion, and transformation of the food system Healthy eating such as more vegetables or more fruits and nuts or less sugar and meat (at least 1 example; additional examples scored as additional information)	Scientific-based diet

a Participants’ group responses were compared with this solution to assess agreement with the expected answers and the number of additional correct pieces of information provided.

b Rating: match with sample solution (0‐2 points).

c Rating: 0.5 points or additional correct information.

d In a German reference sample by Kromeyer-Hauschild et al [29], *childhood obesity* is defined as a BMI >97th percentile. However, international reference samples also use the 95th percentile as a cutoff [30]. Because the scientific papers provided to the students did not contain the sample solution for question 1, the answers “BMI” and “excessive fat accumulation representing a risk to health” were each awarded 1 point.

Beyond quantitative scoring, the group task responses were analyzed qualitatively using an inductive, theme-oriented coding approach based on the principles of data-driven analysis developed by Braun and Clarke [31]. After familiarization with the data, codes were developed and applied to identify recurring themes and patterns. These themes were subsequently reviewed, refined, and documented to complement the quantitative findings.

## Results

### Demographics

Overall, 359 second-year medical students attended one of the 13 course dates. Most participants were women (n=226, 63%). Slightly more students participated in the Winter Semester of the 2023‐2024 academic year than in the Summer Semester of 2024. An overview of the number of students per semester and by gender is provided in Table 2. No further personal information was collected.

**Table 2.** Overview of participants by semester and gender among second-year medical students^a^.

Semester	Number of courses	Gender		
		Man, n (%)	Woman, n (%)	Total
Summer 2024	7	66 (37.9)	108 (62.1)	174
Winter 2023‐2024	6	67 (36.2)	118 (63.8)	185
Overall	13	133 (37)	226 (63)	359

a Gender was assessed with reference to participants’ first names and does not represent their gender identity.

### Perspectives on Childhood Obesity and Planetary Health Diet

On average, students agreed most strongly with question 1, indicating that they considered it very important to understand and treat childhood obesity (mean 4.66, SD 0.59). Students agreed least with question 2, indicating limited practical experience with children with obesity and their parents during medical training (mean 1.59, SD 1.01). Students also rated adequate professional knowledge about childhood obesity as important (question 4: mean 4.33, SD 0.70) and completely agreed that children with overweight and obesity may be more stigmatized because of their weight (question 3: mean 4.53, SD 0.63). Responses to the Planetary Health Diet items (questions 5‐7) were comparatively lower, with mean scores around 3. An overview of results for questions 1‐7 is provided in Table 3.

**Table 3.** Overview of results regarding perspectives on childhood obesity and the Planetary Health Diet^a^.

Question	Mean (SD)	Total
How important do you personally think it is to understand and treat childhood obesity?	4.66 (0.59)	209
Have you had any (practical) experience with children with obesity/and their parents during your medical training?	1.59 (1.01)	209
Do you think that children with overweight and obesity are more stigmatized because of their weight?	4.53 (0.63)	214
How important do you think it is for doctors and medical staff to have adequate knowledge about childhood obesity?	4.33 (0.70)	210
A Planetary Health Diet can help children with obesity.	3.20 (0.87)	201
I try to eat a climate and environmentally friendly diet (eg, plant-based, regional, seasonal, and organic).	3.42 (0.91)	213
It is important to inform patients about planetary health and the Planetary Health Diet.	3.19 (0.96)	208

a The scale for the questions ranged from 1 (“not important at all/no experience at all/do not agree at all”) to 5 (“very important/a lot of experience/totally agree”)*. *The results are based on a brief Mentimeter questionnaire administered prior to the counseling training.

**Figure 2.**
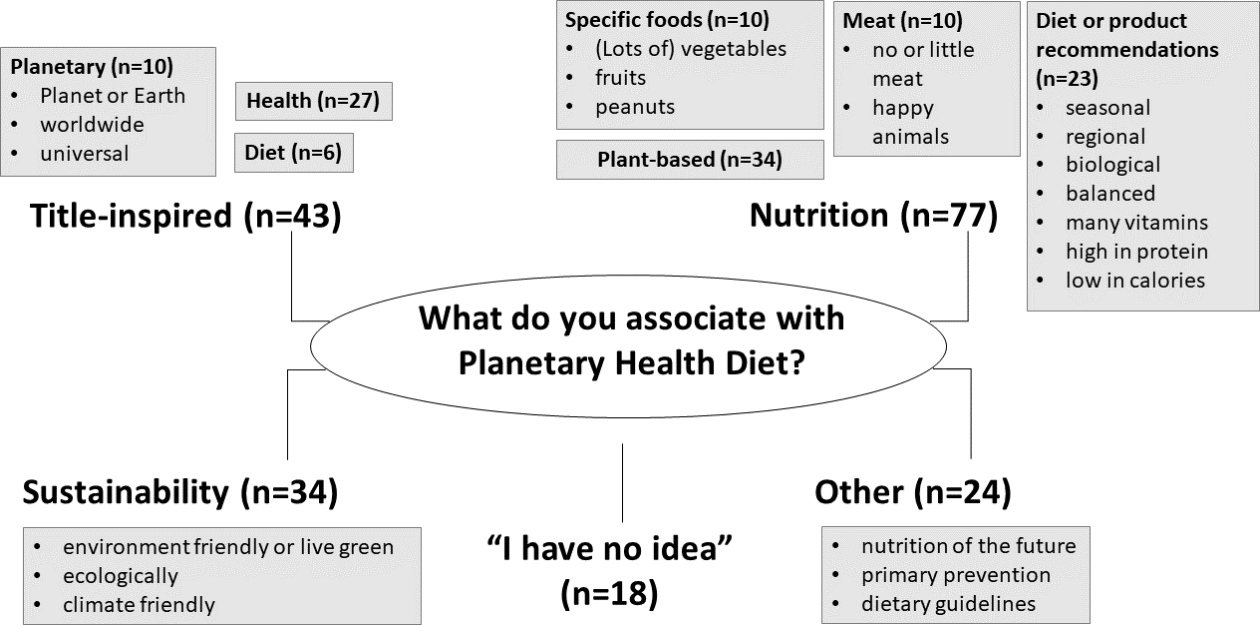
Four main categories of words reported by students in response to an open-ended question about their associations with the Planetary Health Diet. Note: *N*=196, multiple responses per person were possible. Question 8 was open-ended and assessed students’ spontaneous associations with the Planetary Health Diet; responses were categorized into 4 groups: title-inspired, nutrition-related, sustainability-related, and other. The frequency of the words mentioned appears in parentheses.

Question 8 was open-ended and assessed spontaneous associations with the Planetary Health Diet. Students’ responses could be categorized into 4 content groups: “title-inspired,” “nutrition-related,” “sustainability-related,” and “other.” The largest category comprised nutrition-related terms (n=77), including specific foods (eg, “fruit” and “peanuts”), as well as general dietary recommendations; “plant-based” and “(little) meat” were also mentioned. The second-largest category included title-inspired terms (n=43), such as “health,” “worldwide,” and “diet.” Sustainability-related terms formed the third category (n=34). The smallest category included other terms (n=24) that occurred only once and could not be meaningfully categorized. Overall, 18 students reported no associations and responded “No idea.” Figure 2 illustrates the distribution of responses across categories.

### Knowledge Acquisition (Quantitative) Group Task: Evaluation of Group Performance

Table 4 summarizes group performance regarding knowledge acquisition in the task across 4 information-source conditions (ChatGPT, Google, paper, and video); course-level results for all 13 courses are provided in Multimedia Appendix 1. With regard to conformity with the sample solution, the ChatGPT group achieved the highest score (107 points), followed by the video group (100 points), the Google group (97 points), and the paper group (60 points). For additional correct information, the ChatGPT and Google groups showed the highest totals (n=262 each). On average, the ChatGPT and Google groups provided 4.03 units of additional correct information per question and course, whereas the paper group (mean 1.88, SD 1.0) and the video group (mean 1.42) provided fewer additional correct information units.

**Table 4.** Results of the evaluation of the group task^a^.

Group task	ChatGPT	Google	Paper	Video
Conformity with sample solution (points)^b^	107	97	60	100
Total amount of correct additional information	262	262	122	92
Mean number of correct additional information per question and course^c^, mean (SD)	4.03 (1.46)	4.03 (1.26)	1.88 (1.0)	1.42 (1.06)

a Each group completed the task using only 1 assigned information source (ChatGPT, scientific videos, preselected scientific papers, or Google Search) and reflected on content-related aspects, as well as interactional considerations relevant to the counseling interview.

b Scores ranged from 0 to 130 points.

c Medical students were divided into 13 courses, and each group answered 5 questions.

### Knowledge Acquisition and Preparation for Counseling (Qualitative)

In the thematic context analysis, the group performance regarding knowledge acquisition and preparation for counseling was evaluated. The thematic content analysis indicated that the ChatGPT group provided the most detailed responses overall. For question 1, they provided a definition of obesity referencing percentiles as well as age and gender, whereas other groups referred to BMI without specifying percentiles or demographic references. For question 2, the ChatGPT group listed a broad range of causes (eg, genetics, lack of movement, eating habits, environmental factors, psychosocial factors, and medical factors). The Google and video groups primarily reported sociocultural (eg, eating habits and lack of movement) and medical factors; the video group additionally mentioned eating to regulate emotions, while the paper group focused on unhealthy diets, increased calorie intake, and lack of movement.

For question 3, all groups named long-term health risks, including cardiovascular, metabolic (eg, diabetes), orthopedic (eg, musculoskeletal pain), and psychological consequences. The Google, video, and paper groups also provided specific examples of psychological consequences; for instance, the Google group mentioned premature pubertal development, the paper group mentioned bullying, and the video group mentioned bullying and eating disorders.

For question 4, all groups reported balanced nutrition (including fruits and vegetables), healthy fats, a low-sugar diet, and regular physical activity as relevant dietary requirements. The video group additionally referenced using the food pyramid and informing patients about nutrients and food contents. The ChatGPT, paper, and video groups mentioned controlling portion sizes. The Google and ChatGPT groups suggested eating regularly (eg, meals at fixed times). The ChatGPT, Google, and paper groups mentioned unsweetened beverages, and the Google and paper groups reported focusing while eating without distractions.

For question 5, all groups described key aspects of the Planetary Health Diet, including sustainability in nutrition and agriculture, health-oriented dietary patterns, quality rather than quantity, and reducing food waste. All groups also described a predominantly plant-based dietary pattern with an increased intake of fruits, vegetables, whole grains, and nuts. The ChatGPT and Google groups provided more detailed descriptions of the Planetary Health Diet, with the ChatGPT group including more quantitative elements (eg, quantities of food groups) and the Google group focusing more on types of food groups.

### Interactive Behavior With ChatGPT Regarding Counseling

Regarding the counseling, students were allowed to use ChatGPT. Interactive behavior was analyzed for 9 courses; in 4 courses, the chat history could not be saved due to technical reasons. Across all analyzed courses, students entered the worksheet questions into ChatGPT largely as keywords with no or only minor modifications. Five courses asked ChatGPT to shorten the generated answers. Two courses requested a dialogue or role-play, exemplified in Textbox 1. One course requested guidance on how to conduct the role-play and how to communicate information about early childhood obesity. Two courses asked follow-up questions to obtain more detailed information on previously generated answers. One course requested sources for ChatGPT’s statements, and 1 course engaged in brief social interaction (jokes and pleasantries). In 3 chat sessions, no interaction beyond entering the questions was observed.

Textbox 1. **Example of a ChatGPT-generated consultation dialog (translated from German). The dialog illustrates a structured and guideline-oriented counseling approach generated by ChatGPT. While it demonstrates clear communication and evidence-based recommendations, it does not fully reflect the complexity, emotional dynamics, and long-term challenges typically encountered in real-life consultations with families affected by childhood obesity.**

**User: “Write a consultation with the parents of an obese child from a doctor's perspective”**

**ChatGPT:**

**Doctor:** Good day, Mr and Mrs Müller. Thank you for coming in today. I understand that you’re concerned about your son Tim’s weight. Could you tell me a little about what you’ve observed so far?

**Mrs Müller:** Yes, Tim is now 8 years old, and we’ve noticed that he has gained a significant amount of weight over the past few years. We’re worried about his health.

**Doctor:** That’s completely understandable. It’s good that you’re prioritising Tim’s well-being. I’ve reviewed Tim’s weight and height, and based on his body mass index (BMI), he’s above the 95th percentile for his age, which means that he’s classified as obese. Let’s talk about how we can help him to achieve a healthy weight.

**Mr Müller:** That sounds serious. What can we do to help him?

**Doctor:** There are several strategies that we can combine. First, it’s important to make adjustments to Tim’s diet. A balanced diet rich in fruits, vegetables, whole grains, and lean protein will help him to stay healthy. We should cut back on sugary drinks and snacks and instead offer water and healthier snack options such as fruits and nuts.

**Mrs Müller:** That sounds manageable. What about physical activity?

**Doctor:** Regular physical activity is just as important. Children should aim to be active for at least an hour every day. It can be anything that’s fun and keeps him moving—for instance, cycling, playing soccer, or simply playing outside. It would also help if you, as a family, engaged in activities together to motivate Tim.

**Mr Müller:** We could plan more family outings, such as walking or cycling together. What else can we do?

**Doctor:** Another crucial aspect is limiting screen time. That means less time spent in front of the TV and computer and on a smartphone. Screen time should ideally be limited to no more than two hours per day. Instead, you could spend time together playing games or doing creative activities.

**Mrs Müller:** That makes sense. Are there any specific programs or support services that we can access?

**Doctor:** Yes, there are programs that focus on nutrition counselling and physical activity therapy for children. I can provide you with more information about them and arrange for a consultation with a dietitian who can help to create a personalised meal plan for Tim.

**Mr Müller:** That sounds great. We want to make sure that Tim grows up healthy.

**Doctor:** That’s a wonderful approach. It will be important to regularly monitor his progress and ensure that he feels supported. We’ll check his weight and height periodically to ensure that he’s on the right track.

**Mrs Müller:** Thank you, Dr Schneider. We’ll do our best to follow your recommendations.

**Doctor:** You’re welcome. With your support and dedication, Tim has a very good chance of reaching a healthy weight. If you have any questions or need assistance, don’t hesitate to reach out to me.

## Discussion

### Principal Findings

We investigated medical students’ knowledge acquisition regarding early childhood obesity and the Planetary Health Diet across 4 didactic scenarios using different information sources. Across all groups, students were able to identify relevant information on both topics, regardless of the source used. However, differences emerged in the level of detail and elaboration, with the ChatGPT group providing the most detailed responses, followed by the Google group. Overall, students reported limited prior knowledge of both childhood obesity and the Planetary Health Diet, underlining the relevance of addressing these topics early in medical education.

### Importance of Understanding Early Childhood Obesity and the Planetary Health Diet

Although participating medical students reported little prior practical experience with children with obesity and their parents, they strongly endorsed the importance of understanding and treating childhood obesity. Similar findings have been reported previously, emphasizing the need for enhanced obesity-related training to increase confidence and support unbiased, effective care [32-34]. Students also agreed that children with overweight and obesity may experience weight-related stigma [32,33]. In response, physicians require targeted education addressing stigma, respectful language, and patient-centered approaches [34-37]. Previous studies have shown that sensitive communication and patient-centered care are key components of effective obesity treatment [36,37]. Accordingly, medical education should integrate childhood obesity training with a focus on communication and relationship-oriented care.

With regard to planetary health, students neither clearly endorsed nor rejected the relevance of the Planetary Health Diet for treating childhood obesity and expressed ambivalence about informing patients about planetary health more broadly. This uncertainty may reflect limited prior exposure to planetary health concepts in medical education [37], despite students reporting moderate engagement in environmentally friendly dietary behaviors. At the same time, children and adolescents are increasingly adopting dietary practices that align with environmentally sustainable patterns, such as plant-based, regional, and seasonal food choices [38]. These findings suggest that planetary health concepts may benefit from being embedded in concrete clinical counseling contexts to enhance their perceived relevance.

### Knowledge Acquisition and the Role of Different Information Sources

Despite limited baseline familiarity with the Planetary Health Diet, students’ spontaneous associations largely corresponded to its core elements, including plant-based nutrition and sustainability-related aspects [11,12]. When working on the counseling task, all groups were able to retrieve relevant information on childhood obesity and the Planetary Health Diet. However, the ChatGPT and Google groups produced more detailed responses than the paper and video groups.

Students using ChatGPT were able to identify and select generally appropriate information to answer the task questions [39]. Based on the investigation by Arali and Brooks [40], our results showed that using Google or ChatGPT led to reliable answers regarding questions about childhood obesity and planetary health. However, the information generated by ChatGPT could not always be traced to verifiable sources, reflecting a well-documented limitation of ChatGPT (GPT-3.5) [41,42]. In scientific contexts, ChatGPT has been shown to generate references that do not exist [43,44]. Therefore, when integrating ChatGPT into medical education, explicit instruction on critical appraisal, source verification, and transparent referencing is essential [44].

Studies comparing AI tools with Google as an open search engine suggest that AI performs better in terms of general medical knowledge but worse when it comes to delivering medical recommendations [45]. Additionally, students using materials generated by ChatGPT scored significantly higher on post-tests than those who learned through traditional educational videos [46]. Overall, AI systems can enhance learning and achieve strong educational outcomes, although results may vary depending on the specific model used [47]. Unlike traditional, communication-based teaching formats, however, AI and open search tools require critical appraisal skills and careful integration into educational settings to reduce the risk of providing misleading information.

Some students used ChatGPT to generate simulated counseling dialogues. While these dialogues illustrated structured, guideline-oriented communication, they appeared staged and did not fully capture the emotional complexity and long-term challenges typical of real-life consultations. Such outputs may inadvertently convey the impression that childhood obesity can be managed through a limited set of straightforward recommendations. These findings underscore both the opportunities of AI-supported learning and the necessity of explicit guidance on patient-centered, nonstigmatizing communication. For teaching purposes, AI-generated dialogues may therefore be best used as a basis for reflective discussion, highlighting how clinicians might adapt communication to address ambivalence, psychosocial stress, and the chronic nature of obesity, which requires long-term, family-centered, multimodal treatment approaches [48-50].

Compared with ChatGPT, the Google group provided information that was more consistently linked to identifiable sources, facilitating verification. The paper and video groups also produced generally appropriate and reliable information; however, the amount of additional information was limited, likely reflecting both the restricted scope of the materials and the time required to extract relevant content.

### Limitations

This proof-of-concept study has several limitations. First, the information sources differed substantially in scope, with papers and videos offering curated content, while ChatGPT and Google allowed open-ended access. Although this limits direct comparability, it reflects the typical information environments encountered by medical students in educational settings. Second, time constraints within a fixed teaching session may have influenced students’ ability to search, evaluate, and synthesize information. Future studies should, therefore, examine time-on-task and efficiency across different information sources. Third, group work may have introduced variability due to group dynamics. Finally, only a subset of ChatGPT chat logs could be analyzed due to technical limitations.

### Conclusions

Our findings indicate that early, communication-oriented teaching on childhood obesity and the Planetary Health Diet is both feasible and educationally meaningful in undergraduate medical education. Digital tools, including AI-based systems, may enhance students’ knowledge acquisition and elaboration but should not be used without structured pedagogical guidance, critical source evaluation, and reflection on the complexity of chronic care. Teaching these topics within realistic counseling scenarios may support the development of informed, stigma-sensitive, and patient-centered future physicians.

## Supplementary material

10.2196/92644 Multimedia Appendix 1 Overview of the results of the 4 groups broken down for all 13 courses.

## Abbreviations

AI: artificial intelligence
