# Supplementary material for: Communication-Based Teaching on Childhood Obesity and the Planetary Health Diet in Medical Education: Proof-of-Concept Study Comparing 4 Information Sources
Source: JMIR Form Res. 2026 May 8;10:e92644. doi: 10.2196/92644 (PMC13155502; doi:10.2196/92644)
Supplement: Multimedia Appendix 1 [file formative-v10-e92644-s001.docx]

**Multimedia Appendix 1**

Overview of the results of the four groups broken down for all 13 courses

|  |  | Chat GPT | |  | Google | |  | Paper | |  | Video | |
| --- | --- | --- | --- | --- | --- | --- | --- | --- | --- | --- | --- | --- |
| group |  | Conformity with sample solution | Total amount of additional correct information |  | Conformity with sample solution | Total amount of additional correct information |  | Conformity with sample solution | Total amount of additional correct information |  | Conformity with sample solution | Total amount of additional correct information |
| 1 |  | 10 | 26 |  | 8 | 9 |  | 4 | 11 |  | 8 | 4 |
| 2 |  | 7 | 17 |  | 8 | 25 |  | 3 | 5 |  | 9 | 6 |
| 3 |  | 9 | 17 |  | 8 | 16 |  | 6 | 5 |  | 9 | 8 |
| 4 |  | 10 | 21 |  | 7 | 19 |  | 4 | 7 |  | 6 | 2 |
| 5 |  | 7 | 15 |  | 8 | 17 |  | 5 | 13 |  | 9 | 8 |
| 6 |  | 7 | 15 |  | 7 | 25 |  | 5 | 11 |  | 8 | 11 |
| 7 |  | 9 | 32 |  | 9 | 26 |  | 5 | 11 |  | 9 | 3 |
| 8 |  | 8 | 17 |  | 10 | 29 |  | 4 | 6 |  | 10 | 9 |
| 9 |  | 7 | 21 |  | 9 | 25 |  | 4 | 10 |  | 9 | 8 |
| 10 |  | 9 | 18 |  | 5 | 17 |  | 7 | 8 |  | 9 | 6 |
| 11 |  | 9 | 22 |  | 8 | 27 |  | 5 | 13 |  | 5 | 17 |
| 12 |  | 6 | 18 |  | 6 | 15 |  | 5 | 16 |  | 6 | 5 |
| 13 |  | 9 | 23 |  | 4 | 12 |  | 3 | 6 |  | 3 | 5 |

*Note.* Conformity with sample solution (2 points/question)
